# Implementing treatment according to the guidelines is of paramount importance in locally advanced cervical cancer: a real-world study

**DOI:** 10.3389/fonc.2025.1562067

**Published:** 2025-05-08

**Authors:** Ester Jääskeläinen, Henna Kärkkäinen, Jan-Erik Palmgren, Marjut Haataja, Marianne Hinkula, Maarit Anttila

**Affiliations:** ^1^ Department of Gynecology and Obstetrics, Kuopio University Hospital, Kuopio, Finland; ^2^ Faculty of Health Sciences, University of Eastern Finland, Kuopio, Finland; ^3^ Department of Radiotherapy, Kuopio University Hospital, Kuopio, Finland; ^4^ Department of Brachytherapy, Maastro Clinic, Maastricht, Netherlands; ^5^ Department of Gynecology and Obstetrics, Turku University Hospital, Turku, Finland; ^6^ Faculty of Medicine, University of Turku, Turku, Finland; ^7^ Department of Gynecology and Obstetrics, Medical Research Center Oulu, Research Unit of Clinical Medicine, University of Oulu and Oulu University Hospital, Oulu, Finland

**Keywords:** cervical cancer, guidelines, image-guided brachytherapy, definitive radiotherapy, distant metastases, adjuvant chemotherapy, neoadjuvant chemotherapy

## Abstract

**Background:**

External beam radiotherapy with concomitant chemotherapy and image-guided brachytherapy is the standard treatment for locally advanced cervical cancer. This retrospective study compared real-world outcomes with those reported in the literature and evaluated the impact of treatment implementation on the outcomes.

**Methods:**

Medical records of consecutive patients receiving radiotherapy for cervical cancer at Kuopio University Hospital from 2009–2018 were examined. We identified 112 patients with a median age of 53 (27–88) years. The International Federation of Gynecology and Obstetrics 2009 classification stages were IB–IVB, 86% had at least stage IIB disease, and 60% had lymph node metastases. External beam radiotherapy was conducted using intensity-modulated radiotherapy or volumetric modulated arc therapy. Concomitant chemotherapy was administered in 90% of cases. All patients received brachytherapy in magnetic resonance imaging guidance. Seventeen patients received neoadjuvant chemotherapy, deviating from the guidelines, while thirteen patients received adjuvant chemotherapy. The patients were divided into two groups according to how precisely the guidelines were followed, considering the delivery of concomitant chemotherapy, the treatment of lymph node metastases, the radiation dose to the primary tumor, and the overall treatment time. The median follow-up time was 58 months (IQR 35–87), and the primary endpoint was 5-year overall survival.

**Results:**

The mean delivered biological dose to the high-risk clinical target volume was 93.7 Gy. The median overall treatment time was 49 days. Overall survival, disease-free survival, and local control at five years were 60.1%, 57.0%, and 85.7%, respectively. Receiving less than three cycles of concomitant chemotherapy was a negative prognostic factor for overall and disease-free survival. The guidelines were adequately followed in 76.8% (Group 1) and substantially deviated from in 23.2% of cases (Group 2). Differences were observed between the groups in 5-year overall survival (67% vs 39%, p=0.016), disease-free survival (62% vs 42%, p=0.040), and lymph node control (84% vs 61%, p=0.007). Neither neoadjuvant chemotherapy nor adjuvant chemotherapy improved the outcome.

**Conclusions:**

The outcomes in this real-world setting were inferior to those reported in the literature. Implementing chemoradiotherapy and brachytherapy according to the guidelines is essential; deviations from the guidelines could worsen the outcome.

## Graphical Abstract

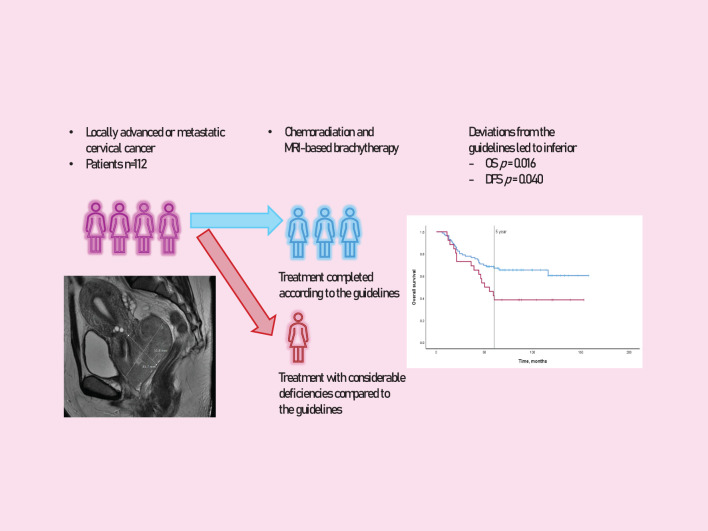

## 1 Introduction

Cervical cancer is often diagnosed when it is already locally advanced and, hence, not suitable for surgical treatment. Nowadays, the curatively aimed standard treatment for locally advanced cervical cancer (LACC) is chemoradiotherapy (CRT) followed by an image-guided adaptive brachytherapy (IGABT) boost to the primary tumor (1–3).

In the last 25 years, there have been significant advances in the treatment of LACC. Chemotherapy used as a radiosensitizer improves overall survival (OS) and disease-free survival (DFS) (4–6). The number of weekly chemotherapy cycles being completed is also essential (7, 8). Radiotherapy technology has improved, and three-dimensional planning is used for external beam radiotherapy (EBRT) and brachytherapy. More precise EBRT targeting has allowed it to deliver larger booster doses to metastatic lymph nodes (LN) (9, 10). For brachytherapy, three-dimensional image guidance with the modern adaptive target concept has been designed, allowing more detailed treatment planning (11, 12).

Studies using modern CRT and IGABT have achieved excellent treatment results for LACC patients. For example, the EMBRACE-I trial had a 5-year OS of 74% (1). However, in real-world clinical practice, not all patients would be suitable for scientific studies, and treatment is not always carried out according to the guidelines.

The implementation of definitive radiotherapy is included in the guidelines published by the European Society of Gynaecological Oncology (ESGO), the European Society for Radiotherapy and Oncology (ESTRO), and the European Society of Pathology in 2018 and updated in 2023 (13). Several factors are included in the treatment, the suboptimal implementation of which may lead to an inferior outcome. These include chemotherapy performed during EBRT (4, 7), a boost to metastatic lymph nodes (9, 10), and the total biological dose from EBRT plus IGABT to the primary tumor (1, 14). In addition, prolonging the overall treatment time (OTT) should be avoided (14, 15). The abovementioned factors are also included in the quality indicators for cervical cancer radiation therapy presented by ESGO and ESTRO (16).

In our unit, LACC patients have been treated with magnetic resonance imaging (MRI)-guided brachytherapy since 2009, using an interstitial component from the beginning. However, compared to the guidelines, there have still been many deviations in implementing definitive radiotherapy in patients.

The aims of this study in a real-world setting were to [1] investigate the long-term outcome with 5-year OS as a primary survival endpoint and to compare the results with the literature and [2] assess how much a deviation from the guidelines affected the outcome.

## 2 Materials and methods

### 2.1 Study design

Gynecological brachytherapy is centralized in Finland into two university hospitals, one of them being Kuopio University Hospital. Records of all patients with primary cervical cancer treated with IGABT in Kuopio University Hospital during 2009–2018 were reviewed retrospectively. The inclusion criteria of the study were as follows: [1] biopsy-proven primary uterine cervical carcinoma, [2] International Federation of Gynecology and Obstetrics (FIGO) 2009 stage IB–IVB, [3] EBRT with or without concurrent chemotherapy, and [4] a high dose-rate brachytherapy boost. Five of 117 LACC patients who fulfilled the inclusion criteria were excluded due to missing follow-up data. Of the study cohort patients, 78% were only referred to our unit for brachytherapy. The ethics committee of Wellbeing Services County of North Savo approved this study.

### 2.2 Treatment

Pelvic MRI and whole-body computed tomography or 18-fluorodeoxyglucose positron emission tomography computed tomography were performed on all patients. All the patients received EBRT with or without chemotherapy at their nearest university or regional hospital. Intensity-modulated radiotherapy was available in referral hospitals before 2009, and the volumetric modulated arc therapy was introduced during the study period. EBRT was performed on the pelvic ± para-aortic LNs. IGABT was performed in four fractions over two weeks immediately after EBRT. The prescription dose was 7 Gy for the high-risk clinical target volume (HR-CTV) per fraction. Our brachytherapy process has previously been reported in more detail (17). The total biologically effective dose from EBRT and IGABT was calculated as equivalent to 2 Gy per fraction. The planning aim, the targeted total dose to 90% of the HR-CTV volume (D90), was 90–95 Gy, with a hard constraint of 85 Gy.

### 2.3 Outcomes

The estimated 5-year OS was selected as a primary endpoint. It was defined as the absence of death from any cause. Secondary endpoints were DFS, local control (LC), and nodal control. These were defined as in the EMBRACE-I study, DFS: absence of any disease event or death from any cause; LC: absence of any recurrent or progressive disease in the cervix, parametria, uterine corpus, and vagina; nodal control: absence of any recurrent or progressive nodal disease in the pelvic, inguinal, or paraaortic region (1). The outcomes were compared with previous studies. To compare our treatment results with the EMBRACE-I study, in addition to the original FIGO 2009, we restaged the patients according to the modified FIGO 2009 classification used in the EMBRACE-I study (eg, patients with paraaortic LN metastases as FIGO IVB) (1).

### 2.4 Implementation of treatment according to the guidelines

The effect of deviations from the guidelines on patients’ outcomes was evaluated by examining four important treatment aspects based on ESGO/ESTRO guidelines: [1] the number of chemotherapy cycles at the time of the EBRT, [2] the radiotherapy boost or surgical lymphadenectomy to the detected LN metastases, [3] the total delivered HR-CTV D90 dose, and [4] the OTT. We assumed that the importance of these aspects to the outcome is roughly equal. The scoring for each factor was assessed as follows: three points if the factor was implemented precisely according to the guidelines; two points if the implementation was partial (concomitant chemotherapy was used, but was not implemented required 5 times; the hard constraint of the HR-CTV D90 dose was met, but not the planning aim; OTT was prolonged less than one week); one point if the deviation was more remarkable than mentioned. The scoring system is presented in **Table 1**. The patients were divided into two groups: in Group 1, the treatment was executed appropriately or with minimal deviations from the guidelines, while in Group 2, there were noticeable deviations compared to the guidelines.

**Table 1:** Scoring system for dividing patients into subgroups according to treatment implementation.

	1 point	2 points	3 points
Concomitant chemotherapy cycles	0	1–4	5–7
Treatment of lymph node metastases (lymphadenectomy or EBRT boost)	No	–	Yes
Overall treatment time	>56 days	51–56 days	≤50 days
Dose delivered to HR-CTV D90	<85 Gy	85 Gy to <90 Gy	≥90 Gy

Points (minimum 4, maximum 12) were calculated to evaluate the quality of treatment implementation:

10–12 points: well-executed treatment, Group 1.

4–9 points: noticeable deficiencies in treatment implementation, Group 2.

### 2.5 Statistical analysis

Descriptive information was calculated as follows: For categorical variables (histology, grade, FIGO stage, LN status, LN treatment, chemotherapy), frequencies were calculated using Pearson’s chi-square test and Fisher’s exact test. For normally distributed continuous variables (tumor size, hemoglobin, number of interstitial needles, delivered dose to LN, HR-CTV D90 dose), means and standard deviation (SD) were calculated using an independent samples 2-sided T-test. For non-normally distributed continuous variables (HR-CTV volume, OTT, reduction of the tumor volume during EBRT), medians and ranges were calculated using the Mann–Whitney U-test. The correlation between the treatment year and the treatment implementation was analyzed using the Spearman correlation test.

The scoring system’s total score for treatment implementation quality could be 4-12. The best cut-off point was found using the minimum p-value method to divide patients into two groups. The Bonferroni correction was applied to account for multiple testing.

The time interval for OS was calculated from the first day of CRT due to the lack of exact data on the initial date of diagnosis for many patients. For DFS, LC, and nodal control, time intervals were calculated from the last day of brachytherapy. All time intervals were calculated until the defined event, or the patients without events were censored on the last follow-up day on which we had information. Survival parameters and the event-free interval were calculated using the Kaplan-Meier method, and a comparison between groups was made using a log-rank test.

Statistical analyses were performed using IBM SPSS software, version 29. Statistical significance was defined by *p* < 0.05.

## 3 Results

This study included 112 cervical cancer patients treated with EBRT with or without concurrent chemotherapy and MRI-based brachytherapy. The median age at diagnosis was 52.5 years, 86% of the cases had at least FIGO 2009 stage IIB disease, 60% were LN positive, and the mean tumor size was 5.6 cm. The baseline characteristics of the patients and their tumors are presented in **Table 2**. The median follow-up was 58 months (IQR 35–87).

**Table 2:** Baseline characteristics of the patients and tumors.

Variable	n = 112	
Age, years	52.5 (27–88)	
Histology		
Squamous cell carcinoma	89 (79.5%)	
Adenocarcinoma	21 (18.8%)	
Other	2 (1.8%)	
Grade		
1	11 (9.8%)	
2	49 (43.8%)	
3	33 (29.5%)	
Missing	19 (17.0%)	
FIGO 2009 stage	Original	Modified^a^
IB1	6 (5.4%)	3 (2.7)
IB2	6 (5.4%)	6 (5.4%)
IIA1	2 (1.8%)	2 (1.8%)
IIA2	2 (1.8%)	2 (1.8%)
IIB	70 (62.5%)	60 (53.6%)
IIIA	4 (3.6%)	2 (1.8%)
IIIB	11 (9.8%)	9 (8.0%)
IVA	2 (1.8%)	2 (1.8%)
IVB	9 (8.0%)	26 (23.2%)
Tumor size, mean (SD), cm	5.60 (1.75)	
Nodal status		
N0	45 (40.2%)	
Pelvic nodal metastasis only	47 (42.0%)	
Para-aortal nodal metastasis	20 (17.9%)	

a – Restaged according to the modified International Federation of Gynecology and Obstetrics 2009 classification used in the EMBRACE-I study^1^.

### 3.1 Definitive radiotherapy and chemotherapy

The EBRT dose was 43.2–50.8 Gy delivered in 25–28 fractions, most commonly (61%) 45 Gy (25 x 1.8 Gy). Of the patients with metastatic LNs, 36% did not receive specific treatment (radiation boost or lymphadenectomy) to the LN. The LN boost was implemented for the first time in 2011; thereafter, it was executed unsystematically until 2016, when the LN boost was used for all patients with LN metastases. Therefore, the implementation of treatment for metastatic LNs correlated with the treatment year (*p* = 0.043). The mean dose of the LN boost, mostly implemented as a simultaneous integrated boost at the time of EBRT, was 57.4 Gy (SD 2.6).

Chemotherapy was concomitantly administered in most cases, but only 52% of patients received five or more cycles of chemotherapy. Seven patients (6%) did not receive chemotherapy, all of whom were treated in the early years of the reviewed time period (2009–2012). In later years, chemotherapy was at least attempted, despite the comorbidities and age. The average number of chemotherapy cycles correlated with the treatment year (*p* = 0.036).

The median volume of the HR-CTV at the time of the first brachytherapy fraction (HR-CTV volume) was 41.2 (8.8–174.8) cm^3^. Interstitial needles were used in 92.9% of cases. The mean delivered biologically effective dose to the HR-CTV D90 was 93.7 (SD 6.2) Gy. The planning aim of 90 Gy for HR-CTV D90 was fulfilled in 75.9% (85/112), and the hard constraint of 85 Gy was achieved in 90.2% (101/112) of cases. Detailed treatment information is presented in **Table 3**.

**Table 3:** Characteristics of the treatment.

Variable	
EBRT mean dose, Gy (SD)	47.0 (2.6)
EBRT technique, n (%)	112 (100%)
IMRT	20 (17.9%)
VMAT	68 (60.7%)
Missing	24 (21.4%)
LN+ patients, n (%)	67 (100%)
EBRT boost to lymph nodes	37 (55.2%)
Lymphadenectomy	6 (9.0%)
Untreated lymph nodes	24 (35.8%)
LN boost technique, n (%)	37 (100%)
Simultaneous integrated boost	26 (70.3)
Sequential boost	6 (16.2)
Missing	5 (13.5)
Lymph node boost mean dose, Gy (SD)	57.4 (2.6)
Whole cohort, n (%)	112 (100%)
Concomitant chemotherapy	101 (90.1)
EBRT without chemotherapy	7 (6.3%)
Missing	4 (3.6%)
5 or more cycles	58 (51.8%)
1 to 4 cycles	43 (38.4%)
Neoadjuvant chemotherapy	17 (15.2%)
Adjuvant chemotherapy	13 (11.6%)
IC brachytherapy	8 (7.1%)
IC/IS brachytherapy	104 (92.9%)
HR-CTV D90 dose, Gy (SD)	93.7 (6.2)

EBRT, External beam radiotherapy; IMRT, intensity-modulated radiotherapy; VMAT, volumetric modulated arc technique; LN+, lymph node metastases; IC, intracavitary brachytherapy; IC/IS, intracavitary plus interstitial brachytherapy; HR-CTV D90 dose, biological dose to 90% of high-risk clinical target volume.

The median overall treatment time for definitive radiotherapy was 49.0 (38–69) days. The recommended treatment time of 50 days was exceeded in 30.4% (n = 34) of patients, but only 6.3% (n = 7) exceeded eight weeks.

Seventeen patients (15.2%) received neoadjuvant chemotherapy (NACT) before radiotherapy. Only four of them had distant metastases. Four patients received NACT due to the original intention to perform a Wertheim operation afterwards (two T1b2N1M0 and two T2bN0M0). In one regional hospital, chemotherapy was carried out systematically for all patients before definitive chemoradiation (n = 7, all FIGO 2009 stage IIB, three of them without LN metastases). The size of the tumor was the reason for NACT in two cases (both approximately 7 cm, still stage IIB, with LN metastases). Most patients received cisplatin and paclitaxel in either a 10- or 21-day cycle. The time interval from the end of NACT to the start of chemoradiation was a mean of 4.9 weeks (2–9 weeks) and a median of 4.0 weeks.

Adjuvant chemotherapy was administered to 13 patients (11.6%) due to a poor response to definitive radiotherapy.

### 3.2 Outcomes

#### 3.2.1 Overall survival

Outcomes according to the FIGO stage are presented in **Table 4**. The 5-year OS rate was 60.1%. Overall, 46 (41%) patients died during the 5-year follow-up, 40 (36%) of them due to cervical cancer. A larger HR-CTV volume was associated with worse OS (*p* = 0.048), and less than three cycles of concomitant chemotherapy led to poorer 5-year OS (27.3% vs 61.9%, *p* = 0.008).

**Table 4:** Outcomes according to the FIGO2009_modif_ stage.

	n	Local failure, n	Nodal failure, n	Any failure, n	5-year local control	5-year nodal control	5-year disease-free survival	5-year overall survival
IB	9	0	1	3	100%	88.9%	66.7%	77.8%
IIA	4	0	0	0	100%	100%	100%	100%
IIB	60	9	12	23	84.7%	78.0%	63.3%	66.4%
IIIA	2	1	0	1	50%	100%	50%	50%
IIIB	9	3	1	5	62.5%	88.9%	44.4%	55.6%
IVA	2	0	0	1	100%	100%	50%	50%
IVB	26	3	8	16	86.1%	68.2%	37.3%	35.9%
Total	112	16	23	49	85.7%	79.0%	57.0%	60.1%

FIGO2009_modif_ – Restaged according to the modified International Federation of Gynecology and Obstetrics 2009 classification used in the EMBRACE-I study^1^.

Five-year OS was 47% for patients who received NACT and 63% for patients without NACT (*p* = 0.23).

Among the patients who had received adjuvant chemotherapy after brachytherapy, recurrence was detected in 10 cases out of 13 (76.9%) vs 39 cases out of 94 (41.5%) without adjuvant chemotherapy (*p* = 0.016), and 5-year OS rates were 23% and 65%, respectively (*p* < 0.001).

#### 3.2.2 Disease-free survival

Five-year DFS was 57%. Recurrence at any site was detected in 49 cases (43.8%). Factors that were found to have a significant connection with cancer recurrence were grade (*p* = 0.014), the LN spread level (no LN metastases, pelvic metastases, para-aortic metastases) at diagnosis (*p* = 0.023), the HR-CTV volume (*p* = 0.044), and less than three cycles of concomitant chemotherapy (5-y OS 27.3% vs 60.6%, *p* = 0.005).

#### 3.2.3 Local control

The actuarial 5-year LC rate was 85.0%. Local recurrence was detected in 16 cases (14.3%). At the 3-month follow-up, a complete response at the primary tumor site was detected in 104 patients (92.9%), and at the 6-month follow-up, a complete response was found in five more patients. Factors affecting LC were the HR-CTV volume (*p* = 0.011), a low reduction of the tumor volume during EBRT (*p* = 0.035), a low hemoglobin level during BT (*p* = 0.043), and a dose of <85 Gy to the HR-CTV (5-y LC 63.6% vs 87.4%, *p* = 0.015).

#### 3.2.4 Nodal control

The 5-year nodal control was 79%. The 5-year LN control was only affected by receiving less than three cycles of concomitant chemotherapy (45.5% vs 82.9%, *p* < 0.001). From 67 patients with LN metastases (either pelvic or para-aortic nodes), 60 had a complete response in LNs 3 months after the treatment. Moreover, all patients who did not have a complete response in LNs at 3 months of follow-up died of cervical cancer.

### 3.3 Deviations from guideline-directed treatment

The patients were divided into two groups based on how closely their treatment process followed the definitive radiotherapy guidelines. The scoring is presented in the Material and Methods section and **Table 1**. We calculated p-values for possible cut-off points from 4–11 versus 12 points to 4–7 versus 8–12 points. The minimum p-value for 5-year OS, DFS, and nodal control were between 4–9 and 10–12 points (0.027, 0.040, and 0.007, respectively), and between 4–7 and 8–12 points for LC (0.010). We used the cut-off point of 4–9 versus 10–12 points, because three of the four outcome indicators agreed with it. Also, the lowest p-value, 0.007, settled on that cut-off point, which was also significant after Bonferroni correction (p=0.035).

In 86 cases out of 112 (76.8%), the treatment was in accordance with the guidelines (Group 1). There were substantial deviations from the guidelines in 26 cases (23.2%; Group 2). The baseline characteristics of the patient groups and their treatment are presented in **Table 5**.

**Table 5:** Baseline data and characteristics of the treatment of the patients in treatment Groups 1 and 2.

Variable	Group 1 n = 86	Group 2 n = 26	*p*-value
Age, years	52.7	59.3	0.019
Tumor mean size, cm (SD)	5.4	6.3	0.008
HR-CTV volume at brachytherapy, cm^3^ (SD)	43.8 (20.5)	50.5 (31.4)	0.21
Histology			0.20
Squamous cell	71 (83.5%)	18 (72.0%)	
Adenocarcinoma	14 (16.5%)	7 (28.0%)	
FIGO2009_org_ stage			0.27
I	9 (10.5%)	3 (11.5%)	
II	60 (69.8%)	14 (53.8%)	
III	11 (12.8%)	4 (15.4%)	
IV	6 (7.0%)	5 (19.2%)	
Nodal status			
N0	43 (50%)	2 (7.7%)	<0.001
Pelvic nodal metastasis only	30 (34.9%)	17 (65.4%)	
Para-aortal nodal metastasis	13 (15.1%)	7 (29.6%)	
Implementation of the treatment			
Concomitant chemotherapy cycles, mean (SD)	4.5 (1.1)	3.2 (2.3)	0.011
EBRT dose, mean (SD) Gy	47.1 (2.6)	47.0 (2.7)	0.92
Lymph node treatment^a^, %	90.7	16.7	<0.001
Lymph node resection, n (%)	12 (14%)	0 (0%)	0.065
Mean dose of lymph node boost, Gy (SD	57.6 (2.6)	56.3 (2.2)	0.37
HR-CTV D90 dose^b^, Gy (SD)	94.8 (5.1)	90.0 (8.1)	0.008
Overall treatment time, days (SD)	48.7 (4.0)	50.8 (6.6)	0.14
Neoadjuvant chemotherapy, n (%)	9 (10.5)	8 (30.8)	0.011
Adjuvant chemotherapy, n (%)	7 (8.1)	6 (23.1%)	0.037

a Lymph node treatment – boost or resection, only for lymph node-positive patients

HR-CTV – high-risk clinical target volume; EBRT – external beam radiotherapy; HR-CTV D90 dose – minimum dose delivered to 90% of the HR-CTV

**Figure 1:**
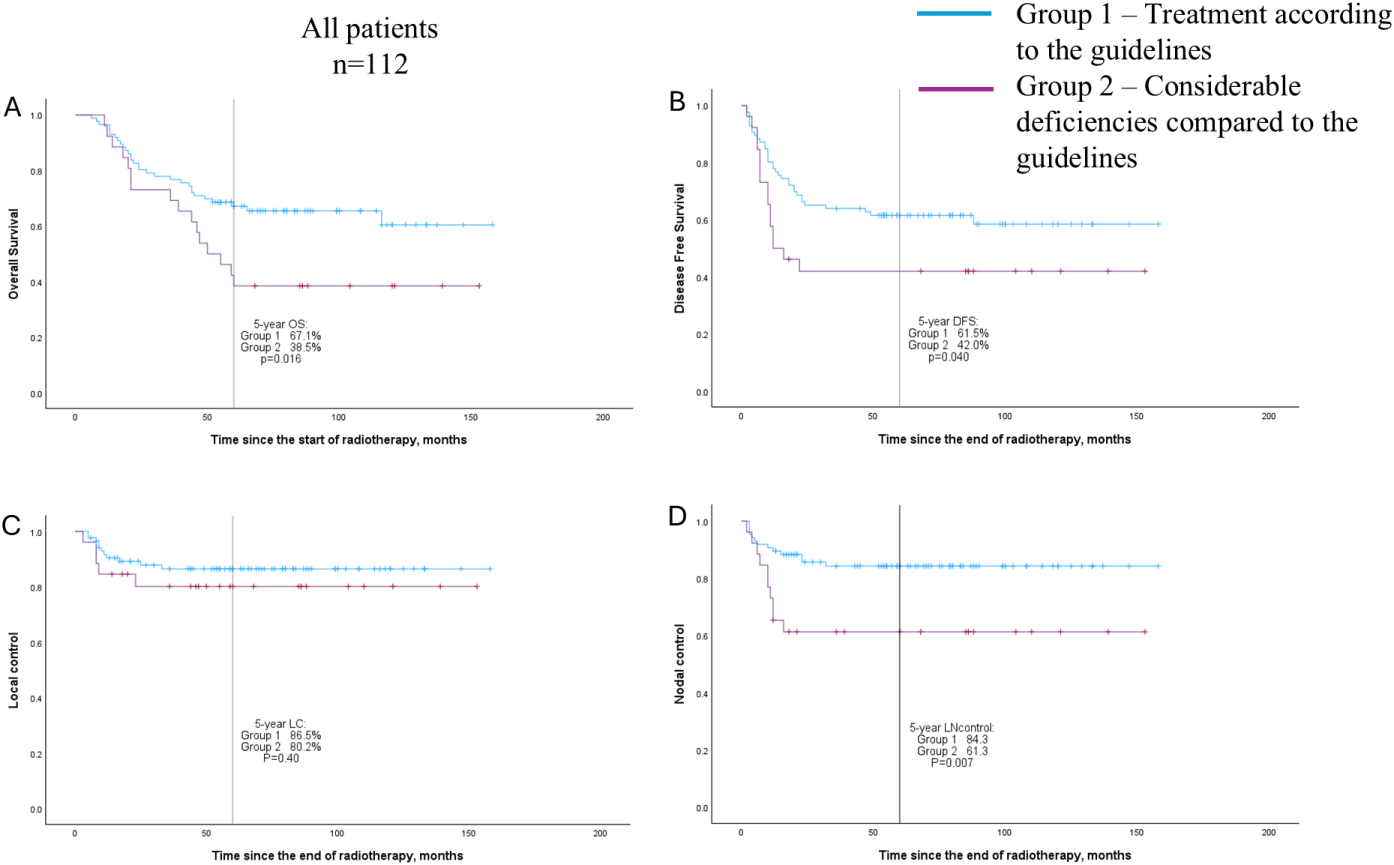
Overall survival **(A)**, disease-free survival **(B)**, local control **(C)**, and nodal control **(D)** curves (Kaplan–Meier method) for patient groups according to the quality of following treatment guidelines; all patients (n = 112).

**Figure 2:**
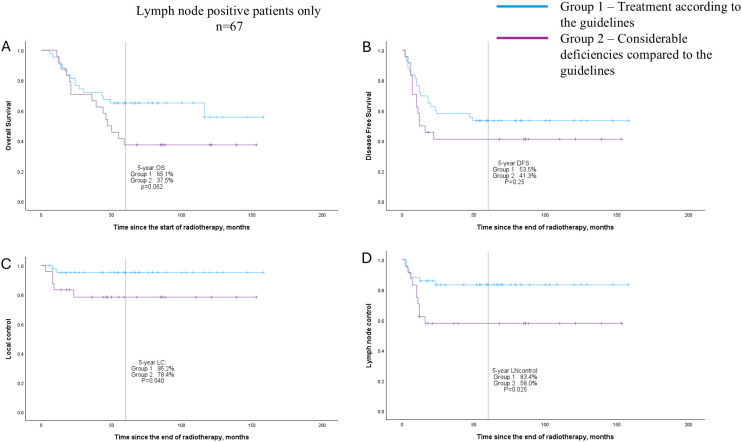
Overall survival **(A)**, disease-free survival **(B)**, local control **(C)**, and nodal control **(D)** curves (Kaplan–Meier method) for patient groups according to the quality of following treatment guidelines; only patients with primary lymph node metastases (n = 67).

In terms of outcome, a significant difference was found in 5-year OS (67.1% vs 38.5%, *p* = 0.016), DFS (61.5% vs 42.0%, *p* = 0.040), and nodal control (84.3% vs 61.3%, *p* = 0.007) between the two groups. We also separately checked patients with LN metastases at diagnosis (n = 67), and a difference between treatment groups was found in LC (95.2% vs 78.4%, *p* = 0.040) and nodal control (83.4% vs 58.0%, *p* = 0.025). See **Table 6** and the Kaplan-Meier curves in **Figures 1**, **2**.

**Table 6:** Estimated 5-year survival according to Kaplan-Meier for Groups 1 and 2.

Variable	Group 1, n = 86	Group 2, n = 26	*p*-value
5-y OS	67.1	38.5	0.016
5-y DFS	61.5	42.0	0.040
5-y LC	86.5	80.2	0.40
5y LNcontr	84.3	61.3	0.007
With lymph node metastases, n = 67	Group 1, n = 43	Group 2, n = 24	
5-y OS	65.1	37.5	0.062
5-y PFS	53.5	41.3	0.25
5-y LC	95.2	78.4	0.040
5-y LNcontr	83.4	58.0	0.025

5-y, 5-year; OS, overall survival; DFS, disease-free survival; LC, local control; LNcontr, lymph node control.

### 3.4 Patients with distant metastases

Our study cohort included nine patients with distant metastases at the diagnosis. Five of the patients were oligometastatic (lung, liver, pelvic bone, or pelvic peritoneum metastases), for which curative chemoradiation, brachytherapy, and local treatment of distant metastases were attempted. Four more patients with distant metastases received NACT and, after a good response, were given EBRT with a brachytherapy boost. All nine patients with distant metastases had received some complementary treatment (NACT, adjuvant chemotherapy, radiation field expanded to pelvic bone metastasis, or stereotactic radiotherapy to liver metastasis). The median follow-up time for this group was 53 months, with an estimated 5-year OS and DFS of 29.6% and 33.3%, respectively. The median OS was 23.9 months (95% confidence interval 5.3–98.7). Two of the patients with liver metastases, treated with stereotactic radiotherapy, and one patient with pelvic bone metastasis, treated during EBRT, were disease-free during the follow-up period.

## 4 Discussion

In this retrospective real-world study on patients receiving radiotherapy for cervical cancer, the 5-year OS rate was 60.1%. The only negative treatment-related prognostic factor for OS was receiving less than three cycles of concomitant chemotherapy. However, we found notable deviations from the guidelines when we assessed the treatment with chemoradiation plus brachytherapy as a whole process. Patients whose treatment had only minor deviations from the guidelines had a significantly better outcome than those who had major deviations in the implementation of the treatment (5-year OS 67.1% vs 38.5%, respectively). Moreover, adding NACT or adjuvant chemotherapy to definitive radiotherapy, which is not according to the guidelines, did not improve the outcome in our study cohort.

EBRT was carried out in our cohort with intensity-modulated radiotherapy or volumetric modulated arc therapy according to modern requirements, and the preferred simultaneous integrated boost technique was used for most LN boosters. However, 36% of the LN-positive patients did not receive an EBRT boost for unresected metastatic LNs. Regional LN metastases are an important prognostic factor for LACC (18, 19). It has been established that a larger radiation dose is needed for metastatic LNs than for the area of microscopic spread (10, 20). ESGO/ESTRO/European Society of Pathology guidelines recommend a simultaneous integrated boost to macroscopic pathologic nodes with a dose of up to 60 Gy (13). Compared to a sequential nodal boost, the simultaneous integrated boost improves survival rates (21). In our study, the deficiency in LN boosting occurred more frequently during the early years of the reviewed time period. Initially, there was concern of an excessively high radiation dose to the bowel. The use of the LN boost increased when this benefit was confirmed in several published studies (22–24). Daily image guidance made it possible to reduce the margins and boost the LNs. Booster administration became routine after the EMBRACE-II study protocol was available.

Most of the study patients received concomitant chemotherapy, and just over half received five or more cycles. Over the decades, the role of concomitant chemotherapy and the number of chemotherapy cycles has been repeatedly demonstrated (6–8). Our concomitant chemotherapy use was similar to the EMBRACE-I patients (no chemotherapy: 6.3% vs. 5.2%, respectively, and five or more cycles: 52% both) (1). The earlier RetroEMBRACE study included more patients (23%) who did not receive chemotherapy (25).

Brachytherapy in our patients was carried out carefully according to GEC-ESTRO and the EMBRACE study recommendations (11, 12). In our cohort, more interstitial component was used (93% vs 28% of cases) (14), and a higher HR-CTV D90 dose (94 Gy) was achieved than in EMBRACE-I patients (90 Gy) (1). Only 10% of our patients received less than 85 Gy to the HR-CTV D90, while in EMBRACE-I, the proportion of patients was 25% (1).

In our study, the median OTT was 49 days, which is according to the guidelines. Evidence shows that prolonging the OTT impairs the outcomes (14, 15). As Parisi et al. state, although prospective randomized trials on the importance of OTT do not exist, it is generally accepted that delays in radiotherapy should be avoided whenever possible (26). Recommendations for the OTT vary from 7 to 8 weeks. Less than a third of our patients exceeded the generally recommended 50 days, and approximately 6% exceeded eight weeks.

### 4.1 Outcomes compared with literature

The 5-year OS rate of our patients was 60%. This is comparable to other retrospective studies that have used definitive radiotherapy with MRI-guided brachytherapy. For example, 5-year OS was 65% in a Swedish study (27), 65% in a Belgian study (28), and 54–64% in a Thai study (29). Furthermore, the retrospective observational multicenter RetroEMBRACE study, conducted during the IGABT’s development phase, recorded a 5-year OS rate of 65% (2). However, our outcome is inferior to the prospective reference study EMBRACE-I, in which a 5-year OS rate of 74% was reported. Similarly, comparing 5-year DFS and LC, our patients’ Kaplan-Meier estimates were 57% and 86% compared to 68% and 92%, respectively, in EMBRACE-I patients (1).

Compared with the guidelines, the most significant deficiencies in our cohort were in receiving concomitant chemotherapy and boosting the metastatic LNs. Receiving less than three cycles of concomitant chemotherapy was associated with inferior OS, DFS, and nodal control. In addition, a HR-CTV D90 dose of less than 85 Gy was associated with lower local control. Our negative prognostic factors do not explain the inferior outcome compared to the EMBRACE-I study, because our use of concomitant chemotherapy was similar, and we fulfilled the planning aim of 85 Gy more often than in the EMBRACE-I study.

Comparing our study cohort with the EMBRACE-I study, our patients had a more extensive spread of cancer (LN metastases 60% vs. 52% in EMBRACE-I patients, fewer FIGO stage I–IIA patients and more stage III–IV patients, and some patients with distant metastases) (1). Compared to the RetroEMBRACE study, our cohort also included more high-stage patients and more LN metastases (60% vs. 42%); moreover, the mean size of the primary tumor was larger (56 mm vs. 47 mm) at diagnosis (25). In addition, during the brachytherapy, our patients had a larger median HR-CTV volume than in the EMBRACE-I and RetroEMBRACE studies (41 cm^3^ vs. 28 cm^3^ and 30 cm^3^, respectively). However, it has been suggested that this is a surrogate for the response during CRT (25), so it depends not only on the characteristics of the tumor but also on correctly scheduled, high-quality initial treatment.

In the comparison according to the stage, our outcome was still worse, specifically in the more extensive cases, although for stages IB and IIA the outcomes were as good or better (1). The explanation for the inferior outcome may partly lie in the more extensive spread of cancer in our cohort, because we accepted for treatment all referred patients, regardless of the challenges in the implementation of brachytherapy. For example, our cohort included a patient who had previously performed a subtotal hysterectomy for a benign reason, two patients with fistulas that developed during the final days of external beam radiotherapy (one of them vesicovaginal fistula over 2 cm, one small urethral-vaginal fistula), and nine patients with distant metastases. Not all of the cohort patients would have met the inclusion criteria of trials. Nevertheless, the more extensive spread of cancer in our cohort cannot be the only explanation for the inferiority in survival. Deficiencies must still be found in the treatment, considering that CRT with IGABT is a complex and sometimes challenging treatment with several components known to affect the outcome.

### 4.2 Effect of implementing treatment according to the guidelines on the outcomes

We hypothesized that deficiencies in implementing a single treatment component do not always affect the patient’s prognosis. However, the patient’s outcome worsens if several treatment components are not fulfilled according to the guidelines. We divided the patients into groups according to the quality of the treatment implementation (considering concomitant chemotherapy cycles, treatment of LN metastases, the HR-CTV D90 dose, and OTT). Based on the literature, we assumed that the four aspects in our scoring system are equally important in terms of outcomes. The impact of these on the outcomes is approximately 6-13% in several articles (20, 30–33). Additionally, one week of OTT prolongation has been estimated to mean a 5 Gy dose to the treatment site (14).

Just under a quarter of the patients had essential deficiencies in the implementation of the treatment. We found that these patients have inferior 5-year OS (*p* = 0.016), DFS (*p* = 0.040), and nodal control (*p* = 0.007). Our scoring system allocated points according to whether a patient had received the necessary LN booster. However, not every patient needed an LN booster, which may have distorted the results. We analyzed patients with LN metastases separately. A statistical association between groups was observed for LC (*p* = 0.040) and nodal control (*p* = 0.025).

Patients whose treatment did not follow the guidelines were older (53 vs 59 years, *p* = 0.019), their primary tumor was larger, and they had more lymph node metastases. There were also more adenocarcinoma and FIGO stage III-IV diseases, with a non-significant correlation. Deficiencies in treatment implementation may be due to the patient not tolerating the treatment (for example, cytostatic treatment in older women) or the fact that achieving the planning aim for HR-CTV is more difficult due to a large tumor.

### 4.3 Neoadjuvant and adjuvant chemotherapy

In addition, 15% of our patients received NACT, although 13 of the 17 patients presented without distant metastases and, according to the guidelines, should have received definitive chemoradiotherapy (13). NACT did not improve the outcome of our patients; 5-year OS was 47% and 63% for patients with and without NACT, respectively, the difference being nonsignificant. Although a recently published INTERLACE trial demonstrated a 5-year DFS and OS benefit from using short-course induction chemotherapy before CRT and brachytherapy, unfortunately, the radiotherapy in the INTERLACE trial was not implemented according to modern standards (34). Lindegaard et al. have presented a doubt that neoadjuvant chemotherapy will prolong the overall treatment time, reduce compliance with concomitant cisplatin, increase the treatment cost, and may lead to increased overall morbidity (35). According to ESGO/ESTRO, the time between referral to the center and the initiation of primary radiotherapy treatment should not exceed 6 weeks (16). For our patients, the median interval between NACT and CRT was 4 weeks. The INTERLACE trial also emphasized a short time between NACT and CRT.

Adjuvant chemotherapy after definitive radiotherapy was administered in 12% of our patients. In most cases, this was due to primarily widespread cancer and, in addition, a poor response to CRT and IGABT. The outcome of patients who received adjuvant chemotherapy was still significantly worse than the others: 5-year OS was 23% vs 65%, respectively. This is in line with the finding presented earlier in the randomized OUTBACK trial that adjuvant chemotherapy does not improve the outcome of LACC patients treated with CRT (36). Modern anticancer drugs are expected to benefit these patients in the future. For example, promising results have recently been published for the anti-PD-1 monoclonal antibody pembrolizumab administered with and after definitive radiotherapy (37).

### 4.4 Patients with distant metastases

Three of nine patients with distant metastases were still disease-free after more than four years of follow-up, with a 5-year OS of 29.6%. FIGO stage IVB patients in our cohort were either oligometastatic or patients who responded well to NACT, and all received complementary treatment in addition to chemoradiation and brachytherapy. Patients with primarily distant metastases are usually excluded from outcome studies aimed at curative goals. However, evidence has recently been presented that cervical cancer patients with distant metastases may benefit from an intensive locoregional treatment with CRT and brachytherapy (38–40).

### 4.5 Limitations and significance

A weakness of this study is its retrospective nature and non-randomized design. The patients were treated during a period of 10 years when the guidelines were still changing; the European guidelines had not been published then, and information about the development steps of definitive radiotherapy had not yet spread to all physicians. For example, it is possible that when the information about the importance of concomitant chemotherapy had not yet spread, it was withheld from the patients with an inferior prognosis (older or in poor condition). This could cause potential bias. Another potential source of bias is that many patients received EBRT at other institutions. So, we lack the exact data on the delineation of the EBRT or information about daily imaging for these patients.

However, technically, the treatment of the patients was carried out according to modern requirements (most patients were treated with intensity-modulated radiotherapy or volumetric modulated arc therapy, most lymph node treatments were performed with SIB, and all patients got MRI-guided brachytherapy), which allows us to extrapolate our data to current practices. This study demonstrates the importance of strictly following the guidelines to bring patient outcomes in real-life clinical practice closer to the results of prospective studies.

### 4.6 Conclusion

In this real-world study, the 5-year OS rate in locally advanced or metastatic cervical cancer patients was 60%. Patients’ outcomes benefited if the treatment was implemented precisely according to the guidelines. This study reinforces the need to follow the guidelines precisely, as otherwise, patients’ real-life prognoses can easily be worse than in prospective trials. If necessary, treatment should be centralized. Especially for patients with a poor prognosis, it is essential to implement all treatment components well. In addition, according to our study, distant metastases do not always exclude the possibility of curative treatment. There is limited retrospective data on intensive radiotherapy in this specific group of patients. A randomized study is required because there might be an opportunity to improve the prognosis of these patients substantially.

## Data Availability

The datasets presented in this article are not readily available because. The raw data supporting the conclusions of this article will not be opened due to ethical reasons. Requests to access the datasets should be directed to ester.jaaskelainen@pshyvinvointialue.fi.
